# Effects of Land-Use and Environmental Factors on Snail Distribution and Trematode Infection in Ethiopia

**DOI:** 10.3390/tropicalmed8030154

**Published:** 2023-03-01

**Authors:** Seid Tiku Mereta, Samson Wakuma Abaya, Fikirte Demissie Tulu, Kebede Takele, Mahmud Ahmednur, Girma Alemu Melka, Mark Nanyingi, Hannah Rose Vineer, John Graham-Brown, Cyril Caminade, Siobhan M. Mor

**Affiliations:** 1 Department of Environmental Health Science and Technology, Jimma University, Jimma P.O. Box 378, Ethiopia; 2 Department of Preventive Medicine, School of Public Health, College of Health Sciences, Addis Ababa University, Addis Ababa P.O. Box 9086, Ethiopia; 3 School of Applied Natural Sciences, Adama Science and Technology University, Adama P.O. Box 1888, Ethiopia; 4 Department of Geography and Environmental Studies, Jimma University, Jimma P.O. Box 378, Ethiopia; 5 Department of Public and Global Health, College of Health Sciences, University of Nairobi, Nairobi P.O. Box 30197-00100, Kenya; 6 Institute of Infection, Veterinary and Ecological Sciences, University of Liverpool, Leahurst Campus, Neston CH64 7TE, UK; 7 Liverpool Veterinary Parasitology Diagnostics, University of Liverpool, Liverpool Science Park IC2, Liverpool L3 5RF, UK; 8 The Abdus Salam International Centre for Theoretical Physics (ICTP), Earth System Physics Department, Leonardo Building, 34151 Trieste, Italy; 9 International Livestock Research Institute, Addis Ababa P.O. Box 5689, Ethiopia

**Keywords:** agroecology, cercariae, freshwater, land-use, snails, trematode

## Abstract

Freshwater snails are intermediate hosts for several snail-borne diseases affecting humans and animals. Understanding the distribution of snail intermediate hosts and their infection status is very important to plan and implement effective disease prevention and control interventions. In this study, we determined the abundance, distribution, and trematode infection status of freshwater snails in two agro-ecological zones of Ethiopia. We sampled snails from 13 observation sites and examined them for trematode infections using a natural cercarial shedding method. A redundancy analysis (RDA) was used to examine the relationship between snail abundance and environmental variables. Overall, a total of 615 snails belonging to three species were identified. *Lymnea natalensis* and *Bulinus globosus* were the dominant snail species, representing 41% and 40% of the total collection, respectively. About one-third of the total snail population (33%) shed cercariae. The cercariae species recorded were *Xiphidiocercaria*, *Brevifurcate apharyngeate distome* (*BAD*), *Echinostome,* and *Fasciola*. Snail species were found in high abundance in aquatic habitats located in the agricultural landscape. Therefore, land-use planning and protection of aquatic habitats from uncontrolled human activities and pollution can be considered as important strategies to prevent and control the spread of snail-borne diseases in the region.

## 1. Introduction

Snails are invertebrate animals of the class Gastropoda found in freshwater and other aquatic habitats around the world. Approximately 5000 species of snails are found to inhabit different habitats worldwide [1,2]. Snails have received considerable attention as they are intermediate hosts for several diseases in humans and animals [3,4]. Snail-borne parasitic diseases, such as schistosomiasis and fascioliasis, give rise to serious risks to human and animal health in many tropical and sub-tropical countries [4]. Millions of people in approximately 90 countries have suffered from parasitic diseases in which snails serve as intermediate hosts [4]. *Lymnaea natalensis* (Krauss, 1848) is an intermediate host of *Fasciola gigantica*, a parasite responsible for fascioliasis in domestic and wild ruminants in Africa [5]. Studies have shown that animal fascioliasis exists in almost all parts of Ethiopia and affects ruminants, with a prevalence of (16–60%) and (14–91%) in coprological and abattoir surveys, respectively. The higher prevalence was recorded in Bahir Dar using coprological findings (60%) and in Gondar via abattoir surveys (91%) [6,7]. Human fascioliasis is classified as a food-borne trematode infection, commonly acquired by ingesting metacercaria encysted on leaves that are eaten as vegetables. A study conducted in north-western Ethiopia indicated that the prevalence of fascioliasis in school children was about 3.3% [8]. The genera Biomphalaria and Bulinus are the most important intermediate snail hosts distributed in different parts of Ethiopia that transmit schistosomiasis [9]. It is estimated that one-third of the population of Ethiopia, 38.3 million people, are either infected or live in schistosomiasis endemic areas [10,11].

The prevalence of snail-borne diseases is largely influenced by land-use changes. According to Lund et al. [12,13], the expansion of agricultural land at the expense of forest cover results in human modification of the landscape that favours the transmission of schistosomiasis. Water resource developments such as irrigation and hydroelectric power are known to contribute to the formation of suitable localized habitats for snails and, consequently, affect the occurrence of snail-borne diseases [14]. In recent years, the expansion of small-scale traditional irrigation schemes in many parts of Ethiopia has created a favorable habitat for snail intermediate hosts and might further increase the risk of snail-borne diseases. Studies have shown that that about 106 million people (13.6% of population at risk of schistosomiasis live in proximity to large impoundments and irrigation schemes [15].

The conversion of a forest ecosystem into agricultural land profoundly affects stream morphology, water chemistry, increased levels of siltation, altered nutrient dynamics, and hydrology [16]. This creates favorable conditions for the survival of snail species and is expected to increase the risk of trematode infection [17]. The widespread deforestation and degradation of natural habitats for crop farming and grazing of animals could also contribute to the loss of aquatic biodiversity including snail predators and competitors [14]. Perrings and Halkos [18] demonstrated that the conversion of forest to agriculture is a major source of biodiversity loss in Africa. Similarly, Halstead et al. [19] indicated that the application of agrochemicals such as herbicides and fertilizers increased the risk of schistosomiasis by decreasing the abundance of predators and competitors that regulate snail populations.

The occurrence and distribution of snail-borne diseases is influenced by the agroecology of the snail habitat [20]. An agro-ecological zone is a type of land that is suitable for different crops and combines data on abiotic and biotic parameters of physiography, soils, vegetation, animals, and human activities with climate [21]. Studies have shown that the presence of stagnated water bodies, marshy pasture land, and free grazing systems is an important determinant for the occurrence of animal fascioliasis in Awash river basin, Ethiopia [21]. On the other hand, climatic factors such precipitation and temperature largely affect the distribution of snail species and occurrence of snail-borne diseases. According to Ahmed et al. [21], high precipitation contributed to the high prevalence of *Fasciola* spp. infection in Ethiopia. Appleton et al. [22] indicated that temperature affects the metabolic processes of both the snail host and the parasite, thus interfering with parasite reproduction within the snail, snail growth, and survival rate. Similarly, Biniam et al. [23] demonstrated that a temperature of 10 °C or above is necessary for both snails to breed and for the development of the parasite.

Although limited studies are available on the snail spatial distribution and trematode infections, location-specific investigation is required to effectively plan and implement snail-borne disease prevention and control strategies. The objectives of this study are as follows: (i) to describe and explore the spatial distribution of snail intermediate hosts, (ii) to investigate trematode infection in freshwater snails, and (iii) to identify the role of land-use and environmental factors on the abundance and distribution of snails and cercarial infection rates. The findings of this study could help to improve the efficiency of the allocation of resources in targeted prevention and control interventions to specific transmission foci.

## 2. Materials and Methods

### 2.1. Study Area

**Figure 1:**
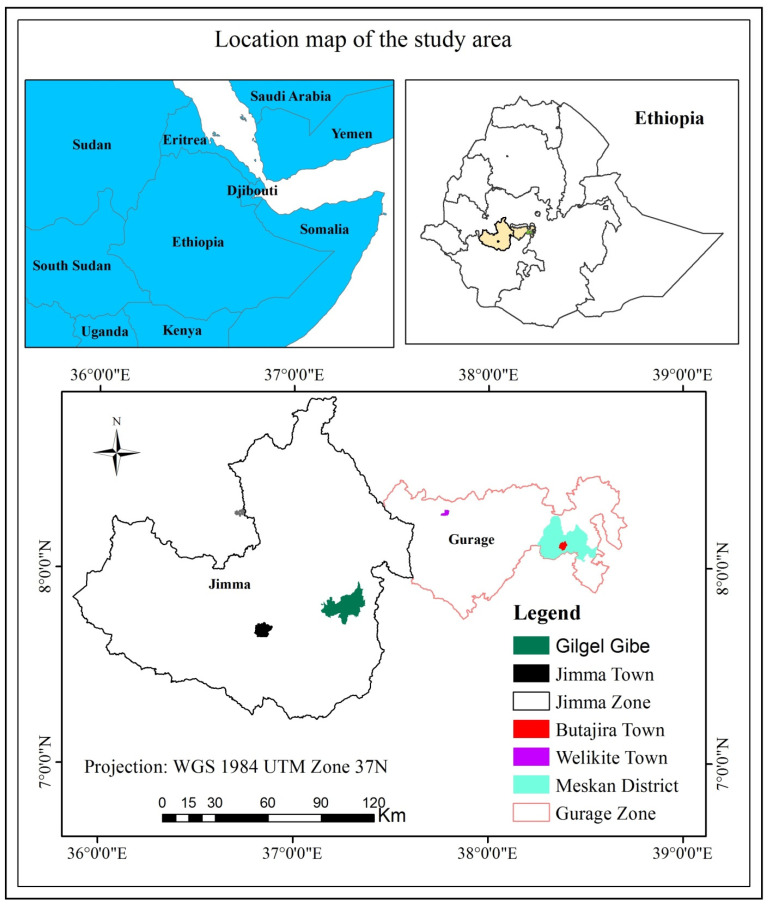
Location of the study area.

This study was conducted in Gilgel Gibe and Meskan districts located in different agro-ecological regions of Ethiopia (Figure 1)). Gilgel Gibe is located in a tepid to cool sub-humid mountain agro-ecological zone, lying between 7°19′ N and 8°12′ N and 36°31′ E and 37°25′ E with a catchment area of 4225 km^2^. The elevation ranges from 1650 to 3000 m above sea level. The mean annual temperature ranges between 15 °C and 22 °C and the mean annual precipitation is between 1800 mm and 2300 mm [24]. The land-use type in this zone includes cultivated land, moderately and perennial crop cultivation, Afro-alpine and sub Afro-alpine vegetation, bush land, shrub land, grass land, wetlands, and perennial river [25]. The Gilgel Gibe I hydroelectric dam is one of the water bodies located in the Gilgel Gibe catchment. This rock-filled embankment dam has a capacity of 917 million cubic meters of waters and generates 184 Mega Watts of electricity [26].

The Butajira site/Meskan district is located the Southern Nations Nationalities and People’s Region (SNNPR) with a tepid to cool sub-humid lake and rift valley agro-ecological zone. It lies between 8°06′ N and 8°52′ N and 37°45′ E and 38°40′ E with a catchment area of 797 km^2^. The elevation of the study area ranges from 1750 to 3400 m above sea level. Annual rainfall ranges between 900 and 1400 mm. The land-use type in this zone includes agriculture, settlement, bushland, shrubland, grassland, and water bodies [25].

### 2.2. Snail Sampling and Identification

A cross-sectional study design was conducted to assess the effects of land-use and environmental factors on snail abundance and trematode infection between January and March 2021. A total of 13 locations were assessed for both agro-ecological zones. The investigated aquatic habitats consisted of streams, rivers, water reservoirs, and natural wetlands. These habitats represented different land-use types so as to depict which forms of land use favor the occurrence of snails and their trematode infection rate. Snail sampling was carried out at each site by handpicking live visible snails and using a scoop net with a wire mesh measuring 1.5 mm on an iron frame (40 × 30 cm) and mounted on a 1.5 m long iron handle for 30 min and placed in ventilated plastic buckets filled with water and vegetation from the sampling site for transport back to the laboratory [27]. The collected snail samples from Gilgel Gibe area were transported to the Laboratory of Environmental Health Sciences and Technology at Jimma University, whereas snails collected from Meskan district were transported to the Butajira Health and Demographic Surveillance System (HDSS) center. Snail species were identified according to the morphological features of the shell [28].

### 2.3. Examination of Cercarial Infection

Collected snails were rinsed in chlorine-free tap water to remove mud and plants. Each snail was kept in a petri-dish of 9 cm diameter containing 20 mL of dechlorinated water at room temperature (25 to 30 °C). Each petri dish was covered with perforated plastics to prevent escaping and to provide good aeration. Each snail was exposed to natural light during the day between 10:00 and 12:00 for 1 h to induce shedding of cercariae [29]. On a daily basis, snails were fed fresh lettuce, the water was changed, and snails were examined for emerging cercariae. Snails that did not shed cercariae in the first hour were monitored for shedding cercariae at 1 h intervals for another 24 h. Snails that did not shed cercariae were kept in glass aquaria in the laboratory and rechecked for cercariae shedding for seven days. The cercariae in the petri dish were stained with 1–2 drops of iodine solution and observed using a dissecting microscope [30]. Cercariae were identified to the genus level based on gross morphological characteristics [31,32]. Snail-species-specific infections were computed as a percentage by taking the number of snails that shed cercariae divided by the total number of snail species examined [33,34].

### 2.4. Land Use, Land Cover, and Spatial Mapping

The Landsat data were obtained from the United States Geological Survey (USGS) website. Sentinel-2 images with a spatial resolution of 10 m were used to assess land-use/land-cover types using Earth resource data analysis system (ERDAS) 2015 image processing software. A supervised image classification technique based on the maximum likelihood algorithm was carried out by creating a training sample and using a spectral signature curve. Accordingly, various land-use classes were created to delineate agricultural land, settlement, bare land, forests, water bodies, and swamp. The classified map was generated using Landsat image for 2020.

A hand-held global positioning system (GPS) instrument (GPS 72H; Garmin Ltd., Olathe, KS, USA) was used to record the altitude and coordinates (latitude and longitude) at each sampling site. The distribution of freshwater snails and sites infected with cercariae along with the land-use types were mapped using satellite images in the geographic information system (GIS) software packages ERDAS 2015 and ArcGIS 10.7 and validated by ground truth points.

### 2.5. Environmental Factors

Water quality parameters were assessed at each observation site. Conductivity, pH, dissolved, oxygen saturation, turbidity, and water temperature were measured in the field using a multi-probe meter (HQ30d Single-Input Multi-Parameter Digital Meter, Hach Company, Loveland, CO, USA). Water depth was measured at each observation site using a graduated stick. Canopy cover within or in the vicinity of the surrounding sampling sites was visually estimated based on the percentage of shade [35].

### 2.6. Data Analysis

The prevalence of infection in the snail population was determined using SPSS version 27. A non-parametric Kruskal–Wallis test was used in STATISTICA 7.0 to determine whether there was a significant difference in environmental factors between the two agro-ecological zones. Comparison between agro-ecological zones was analysed using the Mann–Whitney U-test at a significance level of *p* < 0.05.

In addition, a detrended correspondence analysis (DCA) in CANOCO4.5 for windows, Ithaca NY, USA was used to determine whether a linear (RDA) or unimodal (CCA) type of response was present along environmental gradients [36]. The DCA yielded gradient lengths of less than two and a half standard deviations. In this analysis, snail abundance was considered as a dependent variable, whereas environmental predictors were considered as independent variables. All environmental data except pH were log(x + 1) transformed and standardized. The statistical significance of eigenvalues and snail-species-environment correlations generated by the RDA were tested using 999 Monte Carlo permutations.

## 3. Results

### 3.1. Abundance and Distribution of Freshwater Snail Intermediate Hosts

A total of 615 freshwater snails belonging to three species were collected for 13 sites located in the two different agro-ecological zones. The collected snails include *Lymnea natalensis*, an intermediate snail host for *fascioliasis*, and *Bulinus globosus* and *Biomphalaria pfeifferi*, which are intermediate snail hosts for *schistosomiasis* (Table 1). *L. natalensis* was the most predominant snail species, accounting for 41% of the total collection and collected from 54% of the surveyed sites. *Bu. globosus* was the second most abundant snail species, accounting for 40% of all snails and collected from 38% of the surveyed sites. *B. pfeifferi* was the most frequently occurring species collected from 62% of the surveyed sites (Table 1).

**Figure 2:**
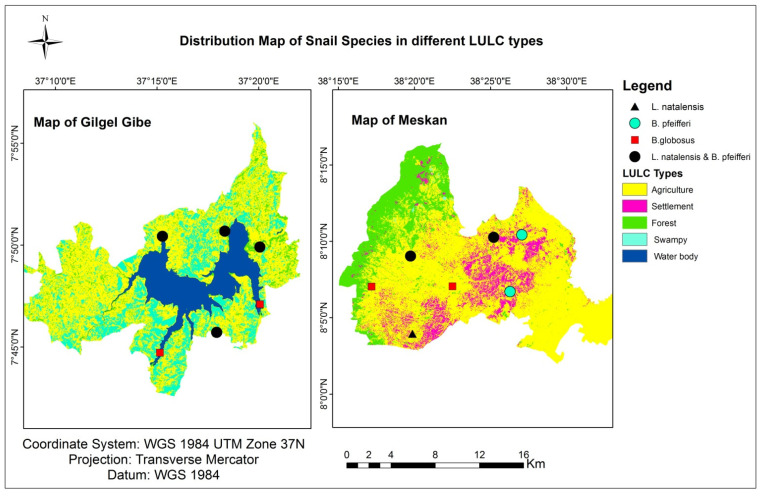
Distribution of snail species in relation to land use.

The distribution of freshwater snail species in different land-use types is shown in Figure 2. The majority of snail species (60%) were collected from water bodies located within the agricultural land use type. The smallest percentage of freshwater snail species (5%) was collected from the forest land-use type. Agriculture is the dominant land-use type in both the Gilgel Gibe and Meskan districts, accounting for 68% and 52.6% of the total surface area, respectively (Table 2).

### 3.2. Cercarial Shedding of Freshwater Snails

A total of four morphologically distinct types of cercariae were identified (Table 3). About 202 (33%) of the snails were releasing cercariae. The cercariae genera recorded were Xiphidiocercaria, Brevifurcate apharyngeate distome (*BAD*), Echinostome, and Fasciola. *L. natalensis* snails shed three types of cercariae and contributed to 61.4% of all infections. *B. pfeifferi* was infected by only *BAD* and contributed to 3.5% of the total infection. The total number of cercariae released was higher in the Gilgel Gibe (59%) district than in the Meskan district (41%).

**Figure 3:**
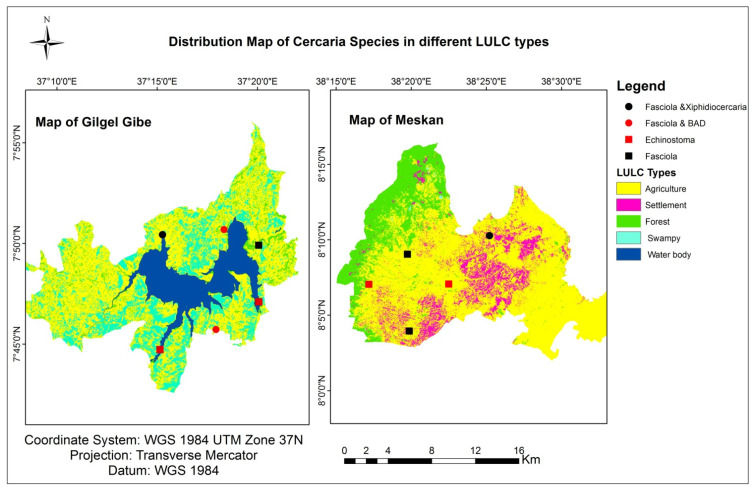
Cercarial infection in relation to land use.

The locations of cercarial infections in different land-use types are shown in Figure 3. Brevifurcate apharyngeate distome (*BAD*) and Xiphidiocercarae were released from snails collected in water bodies located in the agricultural land-use types of the Gilgel Gibe district. These two genera were absent in the Meskan district. On the other hand, Echinostome and Fasciola were released from snails collected from forest and agricultural land-use types in both the Gilgel Gibe and Meskan districts.

### 3.3. Environmental Determinants of Snail Abundance and Trematode Infection

A summary of water physico-chemical variables is given in Table 4. The oxygen saturation level of sampling locations in the Gilgel Gibe area ranged from 13.5% to 110%, with a mean value of 49.72 ± 38.5%. Likewise, sampling sites in Meskan had a mean oxygen saturation of 58.44 ± 34% (Table 4). The highest level of electric conductivity, 506 µS/cm, was recorded in Meskan. However, the variation across all sampling locations was not statistically significant. The highest water temperature was recorded in the Gilgel Gibe area, with mean and range values of 23.88 ± 1 (22.9–25.3) °C, while the lowest was recorded in Meskan, with mean and range values of 21.20 ± 0.98 (19.8–22.4) °C. The highest value of water depth recorded in Gilgel Gibe had a mean value of 0.45 ± 0.9 m, while the lowest water depth was recorded in Meskan with a mean value of 0.20 ± 0.15 m.

**Figure 4:**
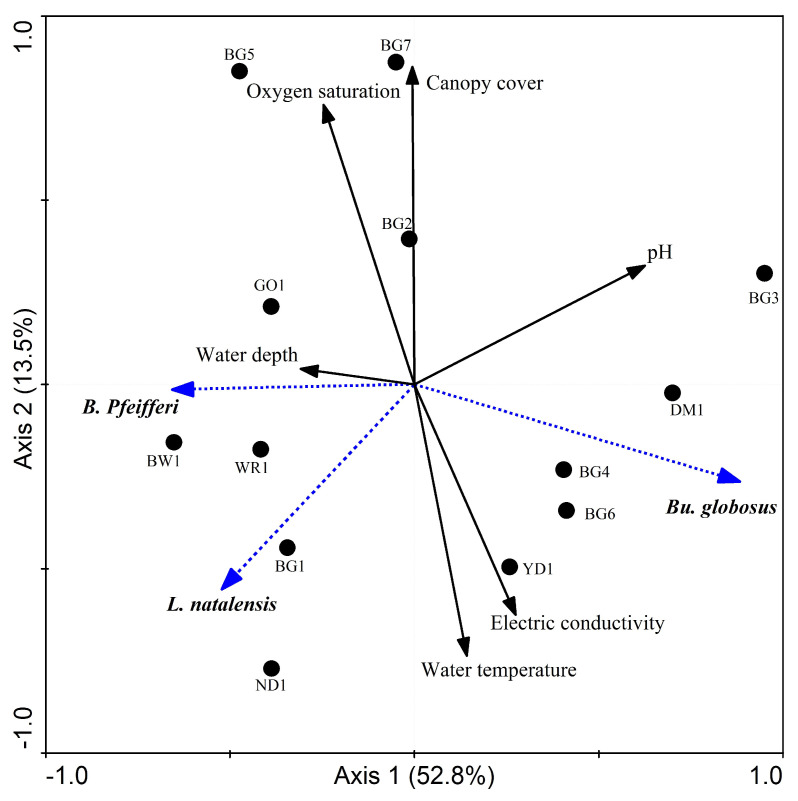
Redundancy analysis (RDA) of snail and environmental variables.

The relationship between snail abundance and the selected environmental variables was found to be significant (*p* < 0.05) for both the first axis and all canonical axes together (Figure 4). The RDA-biplot of snail abundance and environmental variables based on the first two axes explained 66.3% of the variance. The eigenvalues of the first two axes were 0.53 and 0.14, respectively. In this ordination, the snail–environment correlation of the first and second axes was 0.9 and 0.8, respectively. The second axis of the RDA ordination revealed a gradient primarily associated with electric conductivity, water temperature, and canopy cover.

## 4. Discussion

In this study, four types of cercariae and three freshwater snail species were identified. The prevalence of cercarial infection was 33%, which is higher than the estimates in in Ethiopian rift valley regions, where the prevalence of infection was 30.5% [37]. This infection prevalence can be underestimated as the natural cercariae shedding method cannot detect snail prepatent infection rates [38,39]. *Lymnea natalensis* shed three types of cercariae and contributed to 61.4% of the total infection. The occurrence of *Fasciola*-infected lymnaeid snails is an indication of fascioliasis transmission sites in the study area. Studies have shown that animal fascioliasis exists in almost all parts of Ethiopia and affect ruminants with a prevalence of 16–60% in coprological samples and 14–91% in abattoir surveys [5,6]. The high prevalence of trematodes infection in the study area could be attributed to the open grazing system. Animals usually graze open fields and have access to stagnant water from which infection with *Fasciola* metacercariae may likely occur.

The release of human schistosome cercariae (brevifurcate apharyngeate distome) from *B. pfeifferi* could be linked to the transmission of *S. mansoni* infection in the study area. According to Hussen et al. [40], the pooled prevalence of *S. mansoni* in Ethiopia was estimated to be 18%. Likewise, Gebreyesus et al. [41] demonstrated that the prevalence of *S. mansoni* among school children in southern Ethiopia ranged from 11.6% to 54.1%. This study revealed that *B. globosus* was infected by only Echinostome cercariae. Echinostome cercariae are known to cause human echinostomiasis, a disease prevalent in Asia, where insufficiently cooked mollusks, fish, crustaceans, and amphibians are widely consumed [42]. Human echinostomiasis could also be acquired through the consumption of drinking water contaminated by cercariae [43].

The abundance, occurrence, and infectivity of snail species were largely influenced by land-use and environmental factors. The majority of snail species (370, 60%) were collected from water bodies located within the agricultural land. Traditional agriculture is the principal source of livelihood in the study area. Agriculture profoundly affects stream morphology, water chemistry, increased levels of siltation, altered nutrient dynamics, and hydrology [16]. It is also considered as the biggest direct cause of deforestation in the region, mostly for subsistence, that is, growing crops or raising livestock [16,44]. The removal of vegetation for crop farming results in habitat fragmentation and increasing sunlight penetration. This creates favorable conditions for the survival of snail species and is expected to increase the risk of trematode infection [17].

According to Perrings and Halkos [18], the conversion of forest to agriculture is a major source of biodiversity loss in Africa and several species are on the verge of extinction, mainly owing to the wide use of high-yielding varieties. On the other hand, Halstead et al. [19] demonstrated that the application of agrochemicals such as herbicides and fertilizers increased the risk of schistosomiasis by decreasing the abundance of predators that regulate snail populations. The widespread deforestation of natural habitats for crop farming and raising of animals in the study area contributed to the loss of aquatic biodiversity, including snail predators and competitors [14,44]. Mereta et al. [44] demonstrated that aquatic habitats highly disturbed by human activities such as farming, mining, grazing, and waste dumping had a higher abundance of snail species as compared with less disturbed habitats, which harbor a wide diversity of invertebrate predators and competitors. Invertebrate predators belonging to the orders Coleoptera, Odonata, Hirudinae, and Hemiptera suppress the density of snails directly via predation and avoidance of oviposition or indirectly via competition for food resources [45,46].

Previous studies demonstrated that environmental factors such as canopy cover, water physico-chemical quality, and human and animal activities influence snail abundance and infectivity [35,36]. In this study, *B. pfeifferi* were primarily encountered in water bodies with low canopy cover (<20%). The low level of canopy cover likely increases the amount of sunlight reaching the aquatic habitats, thereby increasing water temperatures. Higher temperatures increase the snail metabolic rate, fecundity, and feeding frequency, reducing the duration of the development periods and increasing the number of generations per year and the size of snail populations [47,48].

Among the water physico-chemical variables, oxygen saturation and electric conductivity were important factors that influence the abundance of snail species. The low level of oxygen saturation recorded is an indication of poor water quality. Oxygen saturation was positively correlated with the abundance of *B. pfeiferi* and negatively correlated with that of *Bu. globosus*. On the other hand, the abundance of *Bu. globosus* was higher in habitats with higher water conductivity. Higher conductivity has been associated with organic pollution. Water bodies polluted by human excreta and sewerage from domestic wastes had high levels of electric conductivity as a result of the release of ions as a result of the decomposition process [49]. Overall, the high cercarial infection of snail species in the study area could be attributed to the free grazing feeding system and open defecation practices of the inhabitants. Howell et al. [50] demonstrated that the free grazing feeding system contributed to the high prevalence of bovine fascioliasis in Uganda. Likewise, grazing of animals in boggy pasture and accessibility of animals to water bodies were important determinants for the occurrence of F. hepatica among dairy herds in England [51].

On the other hand, defecation and urination in and around water bodies and open spaces is a common practice in the study area, mainly owing to the absence of sanitation facilities. The Joint Monitoring Program (JMP) of the United Nations Children’s Fund and the World Health Organization estimated that over 28 million people (29%) in Ethiopia are practicing open defecation, predominantly in rural areas [52]. These open defecation and urination practices could be responsible for the release of Schistosoma eggs into water bodies, where they hatch and release miracidia, which enter into snail hosts and release cercariae [46,53]. Infection occurs when humans are exposed to water bodies infested with cercariae released by snail intermediate hosts. Likewise, domestic animals such as livestock, goats, and sheep can be infected with trematodes as water bodies in Ethiopia are commonly used as watering and grazing grounds [15].

## 5. Implication for the Control of Snail-Borne Diseases

Snail-borne diseases can potentially impact society in three ways: (1) they threaten the health of animals, resulting in illness, loss of productivity, and death; (2) they threaten the livelihood of people dependent on livestock as a major source of income; and (3) they cause illness and death in people, which in turn causes additional economic and societal loss. Therefore, a fundamental understanding of the abundance, distribution, and infectivity of the snail intermediate host is very important in order to plan and implement effective snail-borne disease prevention and control strategies, which in turn help to enhance the health of humans, animals, and their shared environment.

## 6. Conclusions

The findings of this study revealed that snail species capable of carrying animal, human, and zoonotic trematodiases occurred in high abundance in aquatic habitats located in the agricultural landscape. Agricultural activities such as grazing and crop farming contributed to habitat fragmentation, as well as changes in ecosystem structure and water quality. Therefore, restriction of free grazing and animal–water contacts, in conjunction with proper management of sanitations, are very important factors to reduce the transmission of snail-borne diseases. Moreover, land-use planning could be an important strategy that can integrate snail-borne disease prevention and control programs for integrated watershed management. Land-use planning implies the judicious use of natural resources such as land, water, biodiversity, and the overall ecosystem to obtain optimum production with minimum disturbance to the environment. Land-use planning balances human and environmental needs, while simultaneously securing ecosystem services and biodiversity [52]. It also enhances productivity and ecosystem integrity regarding the water, soil, plants, and animals within a watershed, thereby protecting and restoring ecosystem services for environmental, social, and economic benefit.

## Figures and Tables

**Table 1:** Abundance and frequency of occurrence of freshwater snails in the Gilgel Gibe and Meskan area, Ethiopia.

Snail Species	Gilgel Gibe		Meskan	
	Abundance	Frequency of Occurrence (%)	Abundance	Frequency of Occurrence (%)
*L. natalensis*	93	67	159	43
*Bu. globosus*	99	33	147	43
*B. pfeifferi*	56	67	61	57
Total	248	_	367	_

**Table 2:** Land-use land-cover (LULC) class area coverage of the Gilgel Gibe and Meskan districts.

LULC Type	Gilgel Gibe		Meskan	
	Area (ha)	%	Area (ha)	%
Agriculture	151,702	52.6	349,075	68
Bare land	67,118	23.3	_	_
Forest	19,772	6.9	100,131	19.5
Water	49,704	17.2	950	0.2
Settlement	_	_	62,480	12
Swampy	_	_	1563	0.3

ha = hectare.

**Table 3:** Cercarial infection of snail species in the Gilgel Gibe and Meskan districts.

Snail Species	Number (%) of Snails Infected by Cercariae			
	*Xiphidiocercariae*	*BAD*	*Echinostome*	*Fasciola*
*L. natalensis*	26	25	0	97
*Bu. globosus*	0	0	47	0
*B. pfeifferi*	0	7	0	0
Total	26	32	47	97

*BAD* = Brivifurcate apharyngeate diastome cercariae.

**Table 4:** Physico-chemical characteristics of snail habitats in the Gilgel Gibe and Meskan districts.

Variable	Gilgel Gibe (n = 6)	Meskan (n = 7)	Z-Adjusted	*p*-Value
Altitude (m)	1674.67 ± 33	2037.14 ± 117	−3.00	0.002 *
Oxygen saturation (%)	49.72 ± 38.5	58.44 ± 34	−0.43	0.58
EC (µS/cm)	307.78 ± 167	337.03 ± 134	−0.57	0.57
pH	7.19 ± 0.4	7.46 ± 0.3	−1.86	0.06
Turbidity (NTU)	66.38 ± 67	105.24 ± 92	−0.57	0.57
Water depth (m)	0.45 ± 0.9	0.20 ± 0.15	2.5	0.01 *
Canopy cover (%)	16.67 ± 16	20.00 ± 9.6	−0.58	0.56
Water temperature (°C)	23.88 ± 1	21.20 ± 0.98	3.00	0.002 *

Asterisk denotes the significance level (* *p* < 0.05).

## Data Availability

The dataset generated and/or analyzed during the present study is available from the corresponding author.
